# Chaperone/Polymer Complexation of Protein-Based Fluorescent Nanoclusters against Silica Encapsulation-Induced Physicochemical Stresses

**DOI:** 10.1021/acs.biomac.4c00689

**Published:** 2024-09-17

**Authors:** Mohsen Akbarian, I.-Ni Chen, Pei-Hsuan Lu, Quynh-Trang Do, Shun-Fen Tzeng, Ho-Hsuan Chou, Shu-Hui Chen

**Affiliations:** † Department of Chemistry, National Cheng Kung University, Tainan 70101, Taiwan; ‡ Department of Life Science, National Cheng Kung University, Tainan 70101, Taiwan; § Marquette University School of Dentistry, Milwaukee, Wisconsin 53233, United States

## Abstract

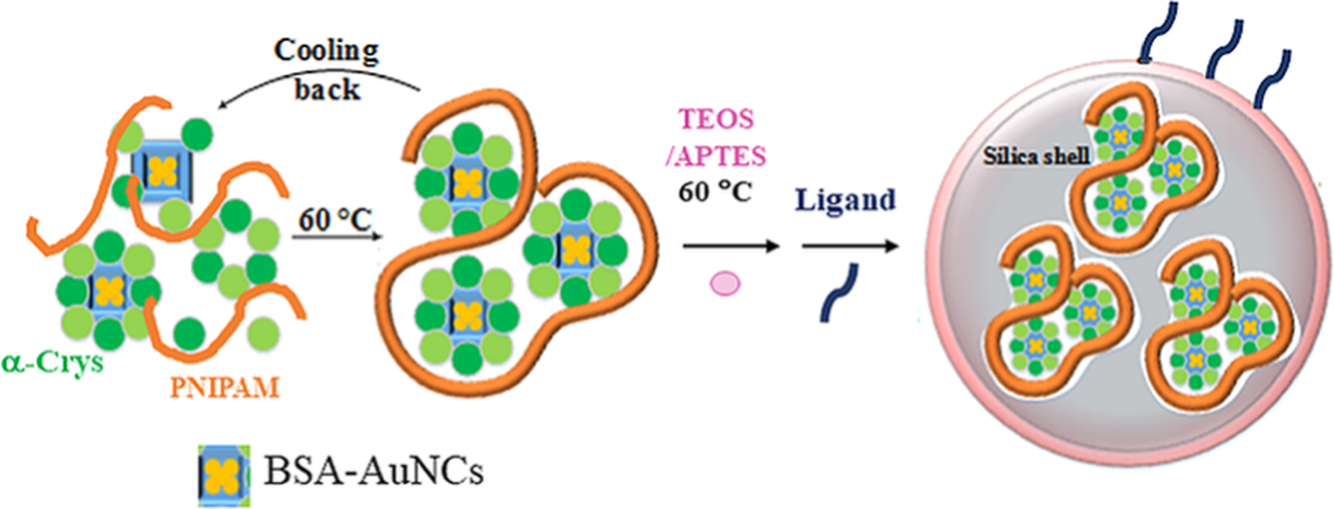

Silica encapsulation under ambient conditions is commonly used to shield protein-based nanosystems from chemical stress. However, encapsulation-induced photo- and structural instabilities at elevated temperatures have been overlooked. Using bovine serum albumin-capped fluorescent gold nanoclusters (BSA–AuNCs) as a model, we demonstrated that chaperone/polymer layer-by-layer complexation can stabilize the template to resist encapsulation-induced fragmentation/reorganization and emission increases at 37 °C or higher temperatures. We first wrapped BSA–AuNCs with α-crystallin chaperones (α-Crys) to gain the highest thermal stability at a 1:50 molar ratio and then enfolded BSA–AuNC/α-Crys with thermoresponsive poly-*N*-isopropylacrylamide (PNIPAM) at 60 °C to shield silica interaction and increase the chaperone–client protein accessibility. The resulting BSA–AuNC/α-Crys/PNIPAM (BαP) was encapsulated by a sol–gel process to yield BαP–Si (∼80 ± 4.5 nm), which exhibited excellent structural integrity and photostability against chemical and thermal stresses. Moreover, targeted BαP–Si demonstrated prolonged fluorescence stability for cancer cell imaging. This template stabilization strategy for silica encapsulation is biocompatible and applicable to other protein-based nanosystems.

## Introduction

Green synthesis of various nanoconstructs using proteins as the capping ligands has been popular in many fields.^1^ Functional groups such as hydroxyl, sulfhydryl, and amine, which are present in proteins, are also known as effective capping agents in plants^2^ including quercetin,^3^ curcumin,^4^ kaempferol,^5^ and tea polyphenol^6^ in herbal compounds. Considering solubility and excretion pathway(s) from the human body, however, the most promising candidates as metal capping agents derived from plants are proteins such as soy protein,^7^ gluten,^8^ and papain.^9^ It should be noted that although the peptides have shown good capping ability,^10^ the half-life and quantum yield (QY) of the peptide-capped clusters are usually not sufficient for medical uses due to limited functional groups necessary to maintain the fluorescence property of gold clusters.^11^ Among the proteins that have the property of forming metal clusters, however, bovine serum albumin (BSA) is the first protein used to synthesize protein-encapsulated gold nanoclusters (AuNCs)^12^ and is considered as one of the top candidates^13,14^ in terms of the cost of production and purification as well as expansion to become universal platforms.^15^

BSA–AuNCs or other protein-capped metal nanoclusters generally exhibit visible to near IR emission with high QY and have been applied for sensing^13,14^ and drug delivery.^14^ However, protein structures that act to shield the metal nanoclusters tend to be disrupted by external harsh conditions such as metals, chemicals, or proteases.^16^ Although quenching property can be applied to sense the presence of these disrupters, such instability leads to a very limited time window for applications in imaging or drug delivery.^17^ In many cases, for diagnostic purposes, it is necessary for the designed drug nanocarrier to have the ability to tolerate high temperatures (e.g., thermotherapy of cancer^18^) and acidic environments.^19^ Since the rate of glucose phosphorylation in cancer cells is higher than oxidation, usually the sugar after phosphorylation to pyruvate accumulates in the cytosol, and instead of entering the mitochondria and entering the oxidation cycle, it converts enzymatically into lactic acid, which leads to the acidification of the microenvironment around the cancer tissue.^19^ Silica encapsulation is one of the common strategies to provide enhanced protection against interrupters to the wrapped fluorophores^20^ and also allow surface functionalization for specific targeting.^15^ However, the protein mobility is constrained to a limited space when encapsulated. Strong physicochemical interactions between protein or silica residues may be induced especially when the temperature increases.^21^ This may affect the protein structure and capping capability, resulting in adverse effects on the wrapped molecules.^16^ It was reported that silica particles increase the fluorescence intensity of rhodamine and malachite green in a size-dependent manner up to a certain critical point and then drop.^22^ The presence of silica layers has increased the fluorescence of gold nanoparticles due to a large local near-electric field induced by dielectric Si particles.^23^ Moreover, some nanoparticles with surface roughness and sharp edges were reported to affect the fluorescence property.^24^ Although higher QY may be gained, such silica-induced instability could lead to inaccurate quantification or negative effects when harmful products are gradually generated and released to the application system.^25,26^ Since most encapsulation methods for protein-based nanoclusters are carried out under an ambient environment,^22^ their physicochemical properties and stability in application environments may differ from those characterized when synthesized. In one case, the gold clusters have been protected by using PVP at first and then have been tried to be thermally stabilized with silica encapsulation. The temperature investigated in this study was considered to be 293 K (approximately 20 °C), which is far lower than the temperature considered for in vivo application (37 °C).^27^ For BSA-encapsulated AuNCs (BSA–AuNCs), silica encapsulation was reported to induce a blue shift in emission due to environmental changes and irreversible aggregation during calcination at high temperatures.^20^ Nevertheless, to the best of our knowledge, there has been no report on improving the stability of encapsulated protein-based AuNCs at biological or higher temperatures.

Natural chaperones that protect proteins from denaturation are excellent candidates for developing stabilization strategies for protein-based nanosystems.^16,28^ In addition to their cost effectiveness and ease of obtaining them, effective chaperone action is important. α-Crystallin (α-Crys) chaperones, which can be effortlessly isolated from the lens of vertebrate eyes, are present in eye lenses at as high as 40% of the total soluble proteins^29^ with anti-inflammatory and antiapoptotic activities.^30^ Additionally, in contrast to many ATP-dependent chaperones, the presence of ATP molecules is not required for αB-Cry chaperone activity,^28^ and also, as they are natural proteins without any artificial modifications, they are safe proteins in terms of toxicity. Past studies have proven that these options can be used as preservatives for medicinal alternatives.^31^ Moreover, due to the many oligomers that α-Crys creates together (roughly 800 kDa α-Crys oligomer),^32^ they can be easily purified by size separation chromatography.^28^ In many studies, it is found that these chaperones can protect their client proteins against a wide range of stresses, of which, temperature stress is among the most important ones.^33^

On the other hand, some nontoxic and biodegradable polymers are good candidates for stabilizing proteins. *N*-Isopropylacrylamide polymer (PNIPAM) has been placed in the acceptable range in terms of its toxicity, so it has been used as a reservoir for medicinal proteins and vaccines in both in vivo and in vitro studies.^34^ As a thermoresponsive polymer, PNIPAM with chain-to-globule transition at moderately high temperatures (lower critical solution temperature, ≈32 °C) potentially wraps the protein complexes when mixed with several proteins.^35,36^ We thus expect that PNIPAM can help the chaperone protein be in better accessibility with client proteins and be easily manipulated to moderate the effect of environmental stresses.

In this study, we will explore the instabilities of BSA–AuNCs induced by the commonly used silica encapsulation method and demonstrate a new template construct and encapsulation method involving a bovine lens α-Crys chaperone and PNIPAM to impart stabilization power. We assumed that the α-Crys chaperone was able to form a thermally stable chaperone/client protein complex, and PNIPAM was able to make an appropriate scaffold for the assembly and increase the accessibility of the chaperone system to target protein (BSA–AuNC) as it undergoes chain-to-globule transition at high temperatures. Moreover, once wrapped by silica polymerization, the constructed nanosystem was expected to be able to protect the embedded fluorophore against a wide range of physical and chemical stresses.

## Experimental Section

The experimental procedures related to chemicals, the extraction and purification of bovine lens α-crystallins, the polymerization of *N*-isopropylacrylamide, BSA–AuNC synthesis, the synthesis of silica-encapsulated BSA–AuNC (B–Si), high-resolution transition electron microscopy, dynamic light scattering (DLS), Fourier-transform infrared (FTIR) measurement, cell culture, functionalization with 17α-ethynylestradiol (EE2) and fluorescence microscopy, as well as Brunauer–Emmett–Teller analyses are prepared as the Supporting Information. The methods that are specifically related to this study will continue here.

### Fabrication of BSA–AuNC/α-Crys (Bα) and BSA–AuNC/α-Crys/NIPAM (BαP)

To calculate the molar ratios, 66.5, 20, and 113.16 kDa were considered as the molecular weights of BSA, α-Crys, and the PNIPAM monomer, respectively. For Bα, the freshly prepared BSA–AuNC (Supporting Information Experiment) was mixed with α-Crys at different molar ratios, kept at 45 °C for 2 h, and cooled back to room temperature. For BαP, the freshly prepared Bα was mixed with PNIPAM (P) at different molar ratios of P to total protein (B + α) in 15 mL of distilled water, incubated at 60 °C for 30 min, and then cooled back to room temperature to form BαP (fresh).

### Silica Encapsulation

A 5.0 mL portion of ethanol was added to the core template (Bα or BαP based on the optimum ratios obtained) solution at 60 °C, and immediately, 170 μL of APTES and 750 μL of TEOS were added dropwise (approximately 50 μL/min) to the solution. After 3.0 h at 60 °C, the precipitate was collected by centrifugation and washed at least three times in distilled water, and the final products, Bα–Si or BαP–Si, were stored in the refrigerator (4 °C) for later use after drying at room temperature. All supernatants were collected to determine the amount of nonreacted proteins and PNIPAM.

### Thermostability

For studying thermostability, the specimens (in phosphate buffer) were sampled so that each solution contained 1.0 mg/mL BSA initially used in synthesizing BSA–AuNC. All the samples were incubated at a specified temperature for the specified time period (60 °C for 7 h, or at 37 °C for 24 h), and the fluorescence spectra (excitation at 365 nm and emission between 550 and 750 nm) were recorded immediately after heating. The spectra of freshly prepared samples (fresh) were also recorded without heating as the control.

### Enzyme and Acid Stability

To check the stability of BαP–Si, all samples were prepared at a final concentration of 1.0 mg/mL BSA–AuNC (4.16 mg of BαP–Si). For enzymatic stability, the sample was incubated with 100 μg/mL of trypsin (1000–2000 U/mg) in 50 mM Tris-HCl (pH 8.0) supplemented with 5.0 mM CaCl_2_ at 37 °C for 20 h. To measure the acidic stability of BαP–Si, the sample was incubated in Tris-HCl at pHs 4.0 and 7.4 for 30 min, respectively. After incubation, the fluorescence spectra of the control samples (bare BSA–AuNC) and test samples were recorded.

## Results

### Instability of B–Si

**Figure 1:**
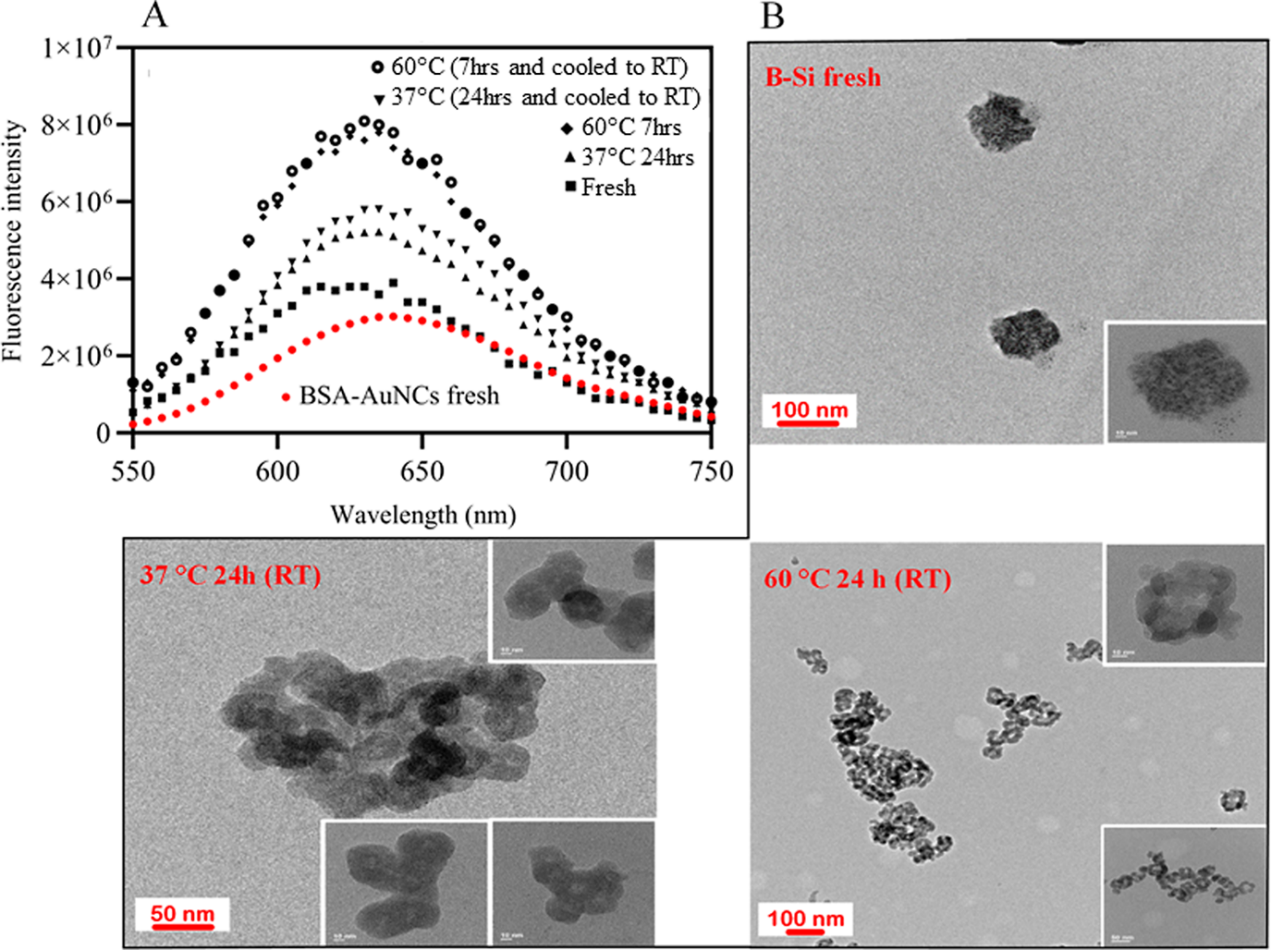
Fluorescence and structural characteristics of B–Si. (A) Fluorescence of B–Si incubated at 37 °C (24 h) or 60 °C (7 h and cooled RT) compared to the freshly synthesized B–Si and BSA–AuNCs (red) at room temperature. The rounded maximum emissions in fresh B–Si and B–Si at 37 °C (24 h), 37 °C (24 h then cooled to RT), 60 °C (7 h), and 60 °C (7 h and cooled to RT) were, respectively, 620, 630, 630, 625, and 625 nm. The fresh BSA–AuNC was also obtained at 640 nm. (B) TEM images of B–Si under specified conditions.

Fluorescence and high-resolution transmission electron microscopy (HR-TEM) assessments were performed to check the photoemission property and structural details of B–Si particles (Figure 1) which were synthesized using reported methods^22^ under ambient conditions. It was seen that B–Si exhibits a blue shift compared to BSA–AuNCs. For BSA–AuNCs, the maximum wavelength (the wavelength at which the maximum emission intensity was recorded) was equal to 640 nm, while for B–S, it was seen to be around 620–630 nm in different incubations. Given the emission intensity compared with BSA–AuNCs, by incubating B–Si at 37 °C for 24 h, the emission increased significantly, almost 2-fold (Figure 1A), showing the impact of temperature on this process. However, the fluorescence of BSA–AuNCs in solution was stable at 37 °C for 24 h (Figure S1), indicating that such thermal-induced photoemission change of B–Si was associated with silica encapsulation. Such increases appeared to be irreversible as the change remained when the temperature was reduced back to room temperature (Figure 1A). Moreover, it did not reach a stable state, and the fluorescence continued to increase as the temperature increased to 60 °C (Figure 1A).

The HR-TEM analyses of these samples showed that the size of each subunit of nanoparticles is fractured, and probably due to these fractures, the connections between the particles are rearranged, and finally, large and irregularly shaped particles are formed (Figure 1B). As protein mobility is expected to be restricted by silica encapsulation, irreversible protein unfolding or enhanced protein–silica interaction may gradually develop to result in fragmentation/reorganization when the temperature increases from room temperature to 37 °C even though it is still below the BSA melting point (63 °C).^37^ It is important to note that gold clusters could not form in BSA when the synthesis was done at 60 °C (Figure S2A), implying that unfolded BSA could lose its wrapping power to protect or stabilize AuNCs. Moreover, FTIR results showed a broad band at 1639 cm^–1^ corresponding to BSA amide I and II stretching (Figure S2B) which were also observed from BSA–AuNC-doped silica nanoparticles,^38^ indicating partial exposure of BSA to the silica surface. It is thus possible that protein–protein or protein–silica interactions through exposed BSA residues may enhance particle aggregation or fragmentation at high temperatures.

When B–Si was synthesized with a 1 M ratio of BSA/Si, the average particle size was equal to 85.3 ± 3.5 nm (Figure 1B), while when this system was synthesized with five times more TEOS, the particle size increased to 106.5 ± 4.6 nm with a thicker silica shell (Figure S3A) and stable emission of B–Si (Figure S3B) at 37 °C for 24 h. However, the emission still increased at 37 °C for 72 h or at 60 °C for 6.0 h (Figures S3B). The data indicated that increasing the amount of TEOS could enhance the stability of Au capping, but changes still occurred at longer incubation times or higher temperatures. Moreover, a blue shift was also seen in fluorescence λ_max_ for all silica-encapsulated samples which can be caused by surface or environmental effects, quantum confinement effect, structural changes in the BSA molecule, and charge transfer interactions.^20,24,39^

### Optimization of BαP Fabrication

In the beginning, we tried to assemble the purified α-Crys proteins (Figure S4) with BSA–AuNC as a chaperone–client complex (Bα) template for silica encapsulation. However, like B–Si, strong protein–silica interaction was induced at 37 °C or higher temperatures when encapsulated by silica (see discussion below). We then sought to protect the Bα complex from silica attack by incorporating thermal-sensitive PNIPAM in the assembly of the chaperone–client system. The BαP complex was then put together in an integrated form through layer-by-layer self-assembly.

**Figure 2:**
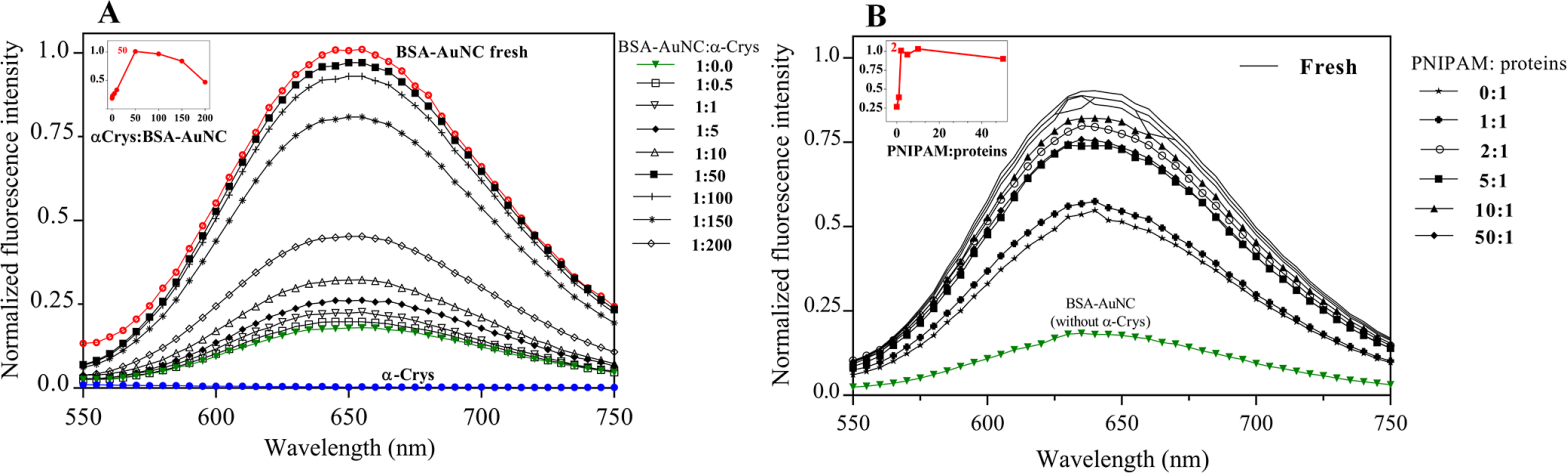
Molar ratio optimization. (A) Fluorescence of the Bα complex at different B/α molar ratios. (B) Fluorescence of the BαP complex at different P/(B + α) molar ratios in which a 1:10 B/α molar ratio was used for Bα complexes. All spectra were recorded immediately upon 60 °C incubation for 7 h. Fresh samples were recorded at room temperature as the control (also see the Experimental Section).

According to previous studies, the subunit exchange of α-Crys subunits (subunit exchanges) increases sharply at >45 °C,^40−42^ and at this temperature, the protection of the client protein is more likely. One of the protection mechanisms by chaperones is the trapping and/or encapsulation of the client protein by the α-Crys complex during subunit exchange.^43^ Therefore, the probability of encapsulating each BSA–AuNC molecule as a client protein by α-Crys subunits is high at >45 °C.^44^ Moreover, it has been reported that the efficiency of chaperones depends on the type of host protein and their concentration.^28,38,45^ Therefore, different concentrations of the α-Crys chaperone and host protein, here BSA–AuNC, were examined to determine the best molar ratio between these two protein sets. As follows, the concentration of BSA–AuNC was considered constant, and the concentration of the chaperone increased incrementally to achieve the best ratio between the chaperone and BSA–AuNC client protein. Figure 2A shows that 50 times more molar ratio of the chaperone had the best effect in protecting BSA–AuNC. Upon further increasing the concentration of the chaperones, the emission decreased (inset of Figure 2A). Therefore, a 50:1 molar ratio of Bα was considered to have the best protection for the photostability of BSA–AuNC.

It is also necessary to obtain the best ratio between PNIPAM and the Bα protein complex at 60 °C which is suitable for the linear-to-globular transition of PNIPAM (and also for chaperone performance and subunit exchange to protect BSA–AuNC fluorescence quenching)^40^ in forming an excellent BαP complex. According to the performance of different molar ratios of Bα (Figure 2A), the 1:10 molar ratio of the Bα system under which thermal resistance power was about 25% of the maximum (under 1:50 ratio) was chosen to monitor whether PNIPAM wrapping can help to improve the stabilization against thermal stress. Concentrations of the polymer were changed incrementally to assemble with Bα (1:10), and the protective effect of the resulting BαP was investigated. As shown in Figure 2B, it was observed that PNIPAM wrapping at 60 °C helps to enhance the protection of the Bα (1:10) system, and a plateau fluorescence was obtained with a 2-fold molar ratio of PNIPAM to the total (Bα) proteins. Increasing the concentration of PNIPAM more than this ratio did not have a significant effect on the efficiency of the chaperone. These results indicated that although α-Crys plays a major role in resisting thermal stress, the polymer was able to enhance the stabilization role of α-Crys. Note that the slight decreases in fluorescence at 60 °C compared to the fresh BαP (Figure 2B) may be attributed to light scattering. Based on these results, the optimum BαP complex was fabricated step by step by mixing a 50:1 molar ratio of Bα at 60 °C and then a 2:1 molar ratio of the PNIPAM monomer to total proteins. Although the emission stability of BαP at such a molar ratio is similar to that of Bα at a 1:50 molar ratio, polymer wrapping played an important role in resisting silica interaction in addition to stabilization enhancement and will be discussed later.

### Characterization of the BαP Core Template

**Figure 3:**
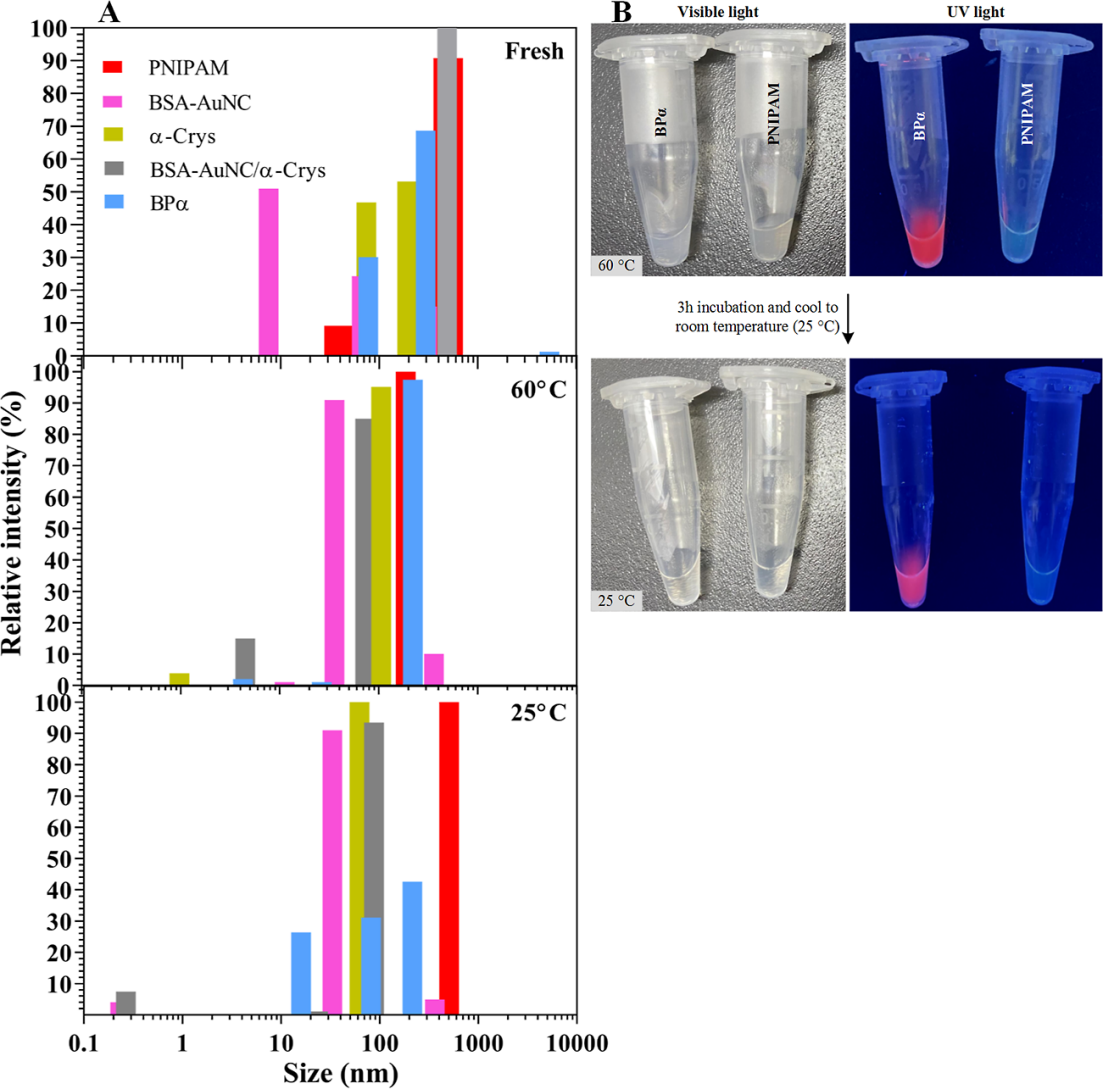
Characterization of core template complexes. (A) Mean size analyses of different samples in three states, fresh, 60 °C, and cooling back to 25 °C. For more simplicity, the average population was represented. (B) Effect of high and low temperatures on the reversibility of the aggregation of PNIPAM and photoemission of BαP under visible room light and UV light.

The hydrodynamic radius of BαP was measured at various temperatures to assess potential interactions among its three components. Each component was tested individually to evaluate their combined effects (see Figure 3A). At room temperature, the PNIPAM polymer was observed in a linear state with a hydrodynamic diameter of 501.9 nm, representing 90.8% of the population. When the temperature was increased to 60 °C, the diameter decreased to 120 nm, suggesting a transition from a linear form to a globular form. Upon cooling back to 25 °C, the diameter increased to 585 nm, indicating reversible changes. The variation between the initial and final linear states (501.9 vs 585 nm) may be attributed to the limitations of the DLS technique in measuring linear molecules. In contrast, for BSA–AuNC, increasing the temperature led to intermolecular aggregation, which did not revert to its original size upon cooling. This irreversibility is likely due to the unfolding of BSA at 60 °C. When BSA–AuNCs were combined with the chaperones at room temperature, a single population with a hydrodynamic diameter of approximately 490 nm was observed. As the temperature rose, new populations with smaller diameters of 73.4 and 4.5 nm emerged. Cooling the sample caused these populations to converge into a single population with a diameter of 87 nm. This complex behavior may result from subunit exchanges between αA and αB Crys and their interactions with BSA–AuNCs.

For BαP, at 25 °C, two main populations with diameters of 74 and 297 nm were observed. At 60 °C, these populations merged into one with a diameter of 220 nm. Upon returning to 25 °C, the 220 nm population fragmented into three distinct sizes: 26, 93, and 220 nm. These observations indicate that each solution was a heterogeneous mixture of free molecules and various assemblies, making it unclear which specific sizes contained the BSA–AuNCs. Overall, the results demonstrate that thermal stress affects BSA–AuNCs irreversibly and that the self-assembly of Bα or BαP dynamically adjusts to protect BSA–AuNCs from thermal stress.

The reversibility of PNIPAM aggregation and its effect on the photoemission of BSA–AuNCs could be observed by the naked eye under visible room light and UV light. As shown in Figure 3B, at first, it was seen that incubation of PNIPAM and BαP samples at 60 °C was accompanied by aggregation of the polymer of both solutions (cloudy under visible light), while the fluorescence property of BSA–AuNC was still preserved in the BαP sample (under UV light). After 3.0 h of incubation at 60 °C and then cooling the samples to room temperature, both samples became a clear solution under visible light, which shows the reversibility of the aggregated state to linear polymer in the presence and absence of the protein complex. However, the photoemission did not change much when cooling back.

### Silica Encapsulation and Characterization of BαP–Si

Before the BαP complex was encapsulated with a silica shell, encapsulation of Bα (without the presence of the polymer) was first examined (Figure S5). In this experiment, we found that Bα encapsulation with silica (Bα–Si) leads to almost complete fluorescence quenching; their incubation at 37 °C for 24 h completely quenched the fluorescence. Their TEM images showed that in some areas, chelated and aggregated structures could be seen, while the presence of spherical silica particles was also evident in them. These indicate that the structure of the Bα assembly was destroyed by silica interruption, losing the protection power of α-Crys to the client protein (BSA–AuNCs). We have tested and found that α-Crys protein could not be used to form fluorescent AuNCs. Therefore, it is possible that α-Crys by itself could not play a role as a capping ligand to stabilize AuNCs.

**Figure 4:**
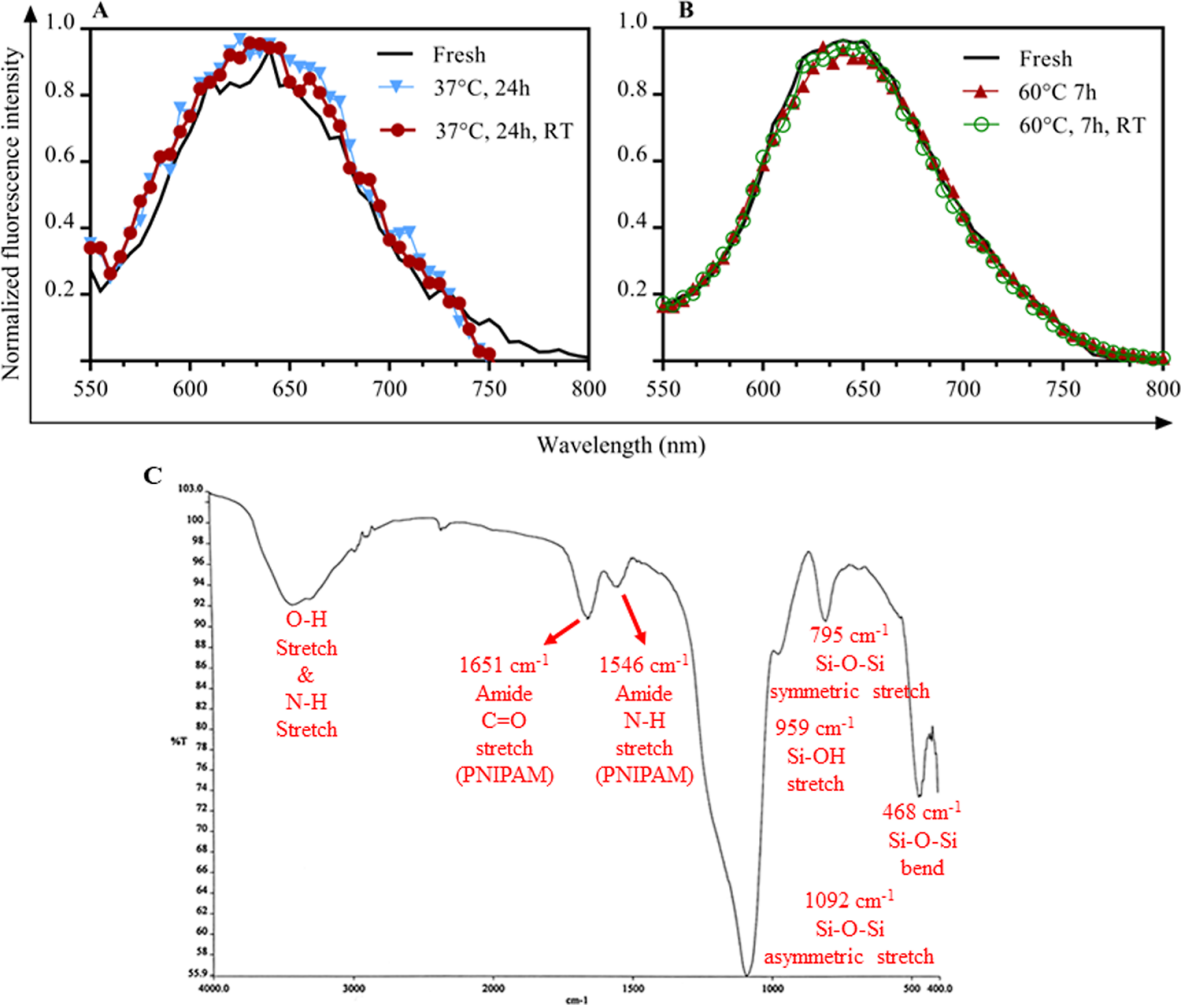
Spectroscopic characterization of BαP–Si. Normalized fluorescence emission of BαP–Si under the fresh condition and after (A) 24 h of incubation at 37 °C and cooling back to room temperature; (B) 7 h of incubation at 60 °C and cooling back to room temperature. (C) FTIR spectra of BαP–Si.

In the next step, silica encapsulation to generate BαP–Si was carried out using BαP with optimized stoichiometry as the core template and at 60 °C under which PNIPAM is in a globular-squeezed form with a one-size (220 nm) population (Figure 3A). When BαP was encapsulated with silica, as shown in Figure 4A,B, the fluorescence emission remained constant with increasing temperature to 37 °C for 24 h or 60 °C for 7 h. It was also seen that cooling the samples back to room temperature does not change the fluorescence emission. Moreover, compared to B–Si (Figure 1), no significant wavelength shift was observed here. In general, compared with other results, the presence of the PNIPAM polymer had a significant effect in preventing fluorescence quenching of Bα upon silica encapsulation. This means that although the presence of α-Crys can be promising for the thermal stability of BSA–AuNC, the presence of a silica shell (which is supposed to act as a barrier against chemicals) leads to quenching, indicating the importance of PNIPAM in keeping photoemission and structure integrity of BαP upon silica encapsulation. Furthermore, FTIR results revealed that two bands at 1651 and 1546 cm^–1^ corresponding to amide C=O stretching and amide N–H stretching of PNIPAM, respectively, were detected in BαP–Si (Figure 4C). These bands were also detected from hollow silica spheres with PNIPAM.^45^ This may also indicate that PNIPAM in BαP–Si was partially exposed to the silica surface, protecting the wrapping protein BSA from exposure as observed in B–Si (Figure S2B).

**Figure 5:**
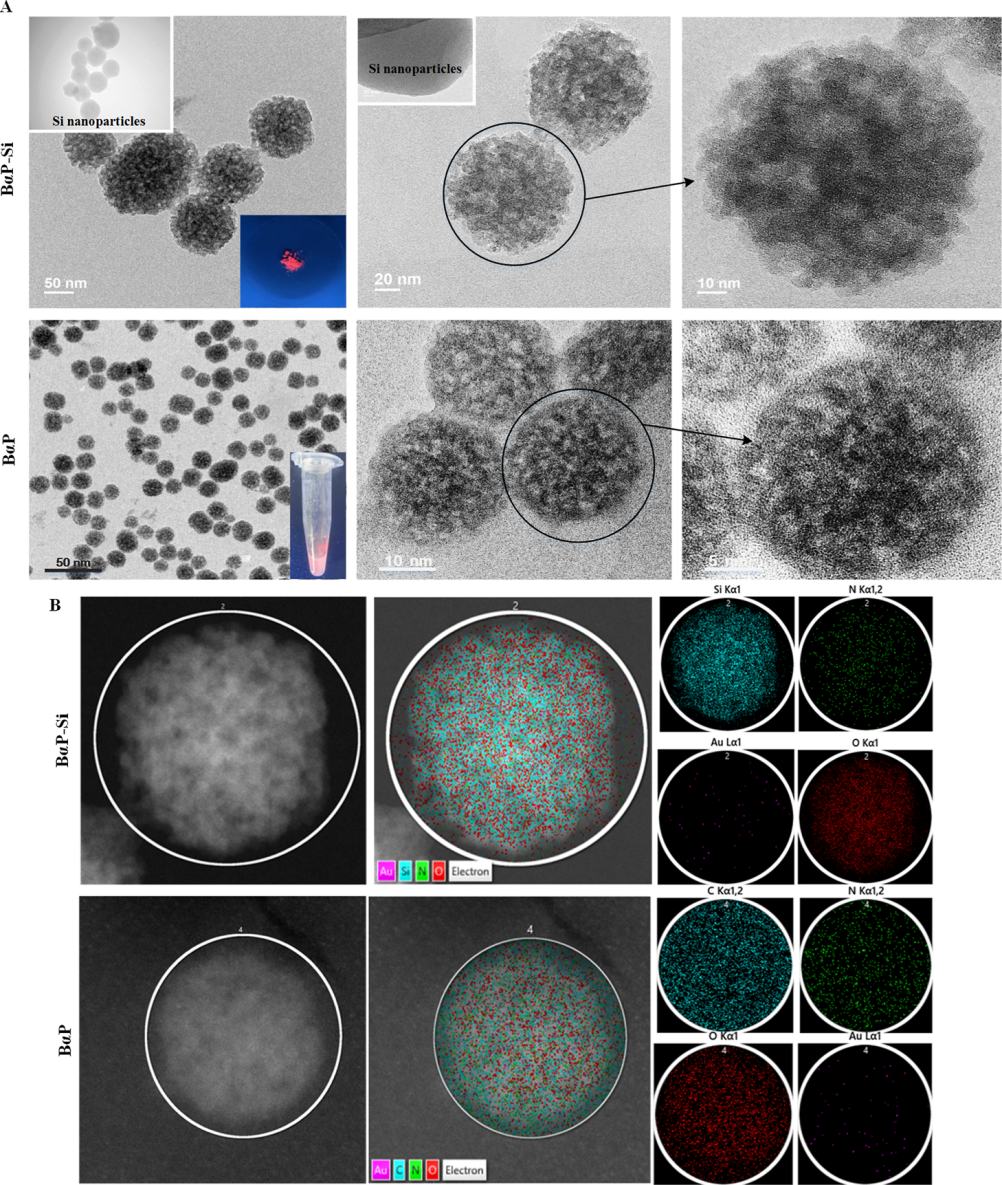
HR-TEM analysis of BαP and finalized BαP–Si nanoparticles.

Different HR-TEM resolutions of BαP–Si and BαP core templates were taken to focus on more details on the samples (Figure 5). On average, the size of BαP–Si was ∼80 ± 4.5 nm, while the BαP core was present at the average size of ∼29.4 ± 2.1 nm. Also, the general surface morphologies of both particles are similar without a clear silica shell. Examining the components of gold, carbon, nitrogen, and silica showed that silica and oxygen atoms dominate the surface; gold atoms have been distributed in the form of several gold clusters in both the PNIPAM and Si particles. These indicated that these particles were wrapped by a thin silica layer. Moreover, unlike B–Si (Figure 1), HR-TEM indicated that BαP–Si was stable under 37 °C incubation for 24 h without significant structural changes (Figure S6).

To estimate the amount of protein loaded in BαP–Si, a Lowry assay was used. According to the standard curve of the fluorescence signal (at 650 nm) versus the amount (mg) of over 100 mg of the total protein used to synthesize BαP–Si, 7.5 mg was not incorporated into the nanostructure. Moreover, after the synthesis of the nanoparticle, in the next step, the possible release of the proteins was checked by the Lowry assay again (Figure S7). Generally, after 55 h of incubation, keeping the sinking conditions, 2.25% of the total amount of protein in the structure was released, and no more was detected after that. Using rhodamine 6G as a reference (Figure S8), the QY for BSA–AuNC was measured to be 5.77%, similar to that reported previously.^13^ B–Si and BαP–Si were measured to be 0.003% and 0.017%, respectively. The decrease in QY after silica encapsulation may be due to light scattering and poor suspension of silica particles. However, the higher QY of BαP–Si compared to B–Si demonstrated another advantage of wrapping BSA–AuNC with αP (BαP) in gaining a higher QY.

### Stress Resistance of BαP–Si

**Figure 6:**
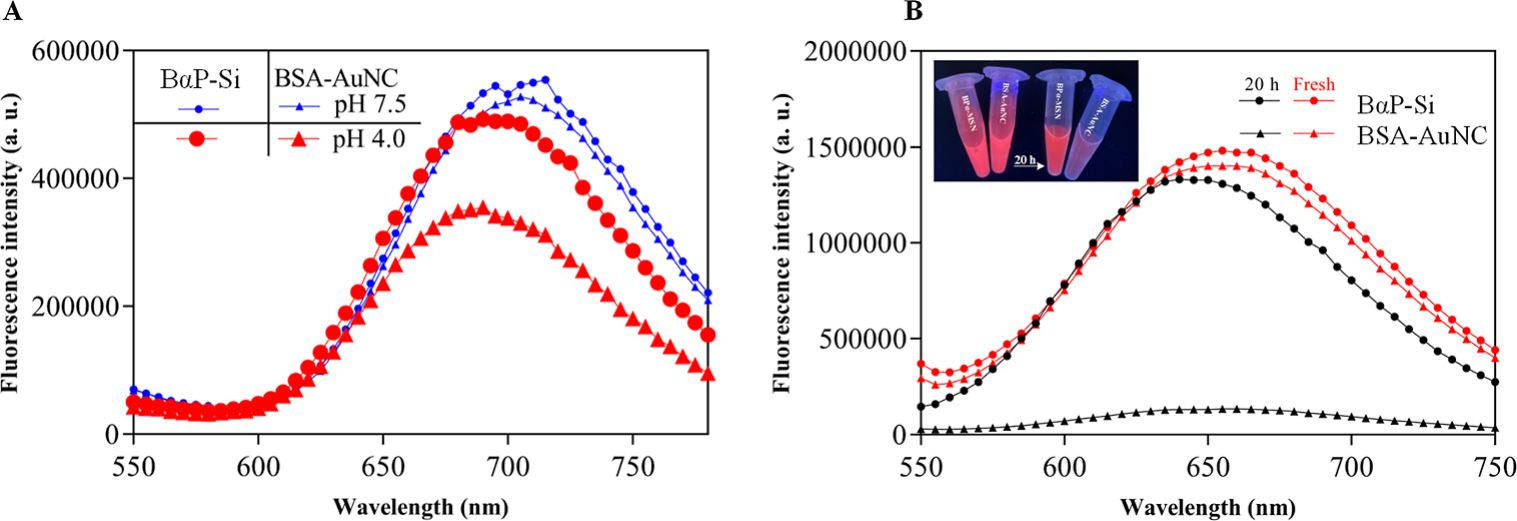
Stability of BαP–Si nanoparticles against (A) acidic pH and (B) presence of 100 μg/mL trypsin.

Regarding the chemical stress tolerance of BαP–Si, the acidic pH has a much less significant quenching effect on BαP–Si compared to that of the bare BSA–AuNC (Figure 6A). Likewise, upon 20 h of incubation of BαP–Si and BSA–AuNC samples in the presence of 100 μg/mL trypsin (Figure 6B), we observed a dramatic drop in control fluorescence, while the BαP–Si nanosystem did not show significant changes.

### Cell Imaging

MCF7 breast cells which express nuclear estrogen receptor α (ERα) were used for cell imaging by functionalizing the surface of BαP–Si with EE2, the binding ligand of ERα.^46^ Moreover, in order to better compare characteristics such as drug specificity and intracellular stability of the nanoparticle, two control nanoparticles, BαP–Si without EE2 functionalization and BSA bound to EE2 (BSA–AuNC–EE2), were used. In previous studies, it has been realized that due to the phagocytosis of cancer cells and the size of silica particles (50–90 nm), after 6 h of incubation,^47^ the cells can absorb these nanoparticles. Therefore, to ensure the specificity of BαP–Si–EE2 particles for MCF7 cells, a 2 h incubation was considered (Figure S9). After the period, it resulted in those particles targeted with EE2 (BαP–Si–EE2 and BSA–AuNC–EE2) entering the cells; nonetheless, the nanoparticle without the EE2 could not enter. Looking at the staining of the nucleus (DAPI staining), and comparing it with the distribution of particles in the cell (fluorescence of BSA–AuNC, the red blots), it was seen that BSA–AuNC–EE2 enters even into the nucleus; however, the BαP–Si–EE2 nanoparticle was able to be traced only to the cytoplasm of the cell. After 24 h of incubation of the samples (Figure S10), it was seen that both targeted and nontargeted BαP–Si samples were present in both the cytoplasm and nucleus; nevertheless, BSA–AuNC–EE2 was quenched under the effect of internal cellular factors. Possible stress factors in cells include proteases, the acidic environment of vesicles, and the presence of radicals and intracellular reducing agents, and these results indicate that BαP–Si–EE2 was able to prolong the imaging lifetime compared to that without encapsulation. Finally, it is important to mention that the BET results (Figure S11 and Table S1) showed that some pores (14 nm in diameter) are detectable in BαP–Si, which may have led to partial exposure of BSA (Figure S2B) or PNIPAM (Figure 4C) observed by FTIR spectroscopy. As mentioned before, the average size of this nanoparticle in fresh form was measured to be around 80 ± 4.5 nm, while when warmed at 37 °C for 24 h, the size was almost stable, 81.2 ± 3.3 nm (Figure S12), indicating the structure stability at the physiological temperature.

## Discussion

In the study of the interaction between BSA–AuNC and the α-Crys chaperone, it was seen that a 50 times higher molar ratio of the chaperone can have the best protective effect for its client protein. The study conducted by Ghosh et al.^48^ showed that by isolating the active domains of αB crystallin (interactive sequences, _73_DRFSVNLDVKHFS_85_ and _131_LTITSSLSDGV_141_), the ratio of 50:1 of the sequences to βH crystallin, as a client model, leads to 30% of protection, while the 10-fold ratio of the sequences to another client model (citrate synthase) resulted in a 90% protection against high temperatures (50 °C), while in many other studies, the optimum ratio between the chaperone and other clients was different; e.g., in a study by Farahbakhsh et al., it was observed that the weight ratio of 6:1 chaperone to insulin B chain (almost equal molar ratio) can provide complete protection^49^ of B chain aggregation. BSA as a large molecule (66.5 kDa) is relatively a hydrophobic client for α-Crys.^50^ Regarding the action mechanism of these chaperones, researchers have so far reached the general view that at high temperatures, the chaperones will bind to some temperature-sensitive domains of client proteins to prevent the binding of unfolded subunits in forming large oligomers.^28^ However, having a dynamic structure is important for better activity of chaperones.^51^ From the stoichiometric studies of different ratios of proteins BSA–AuNC and α-Crys (Figure 2A), we found that limiting such dynamics by increasing the concentration of α-Crys beyond a 50:1 molar ratio will reduce the performance of α-Crys. Controversially, some researchers believe that the chaperones are able to minimize the activation energy for the refolding of unfolded proteins.^52^ Nevertheless, α-Crys complexation played a role in suppressing the thermally induced structural changes of the capping ligand assembly of BSA–AuNC, enabling silica encapsulation at high temperatures (60 °C) without disrupting it. Regarding the structural role of α-Crys at different temperatures in chaperone activity, it resulted in the following: at 62 °C, most α-Crys oligomers transformed to a multimeric molten globule-like state, which is accompanied by drastic changes in the tertiary and quaternary structures, while the secondary structure is almost constant (compared to the structure at 25 °C).^53^ Thereby, these molten structures play a main chaperone role in maintaining the structure of client proteins. Correspondingly, the protective role of α-Crys in BSA–AuNC at a high temperature (60 °C in this study) may be due to the presence of this complex.

According to Figure 2B, PNIPAM wrapping can help enhance the stabilization power of α-Crys as the same protection using the optimum (50:1) ratio was gained with fewer α-Crys (10:1 ratio) in the presence of PNIPAM. Moreover, the 2-fold molar ratio of the PNIPAM monomer to the protein complex (Figure 2B) indicated that most probably each protein unit was covered by two PNIPAM monomers; beyond that, the change of thermal resistance is not significant (reaching a plateau). However, 50 times the PNIPAM monomer appeared to slightly drop the activity (Figure 2B), indicating the undesired crowding effect of the PNIPAM monomer. Woods et al.^54^ stated that the presence of disordered sequences in sHSP causes dynamics in the chaperone structure, but the increase of this dynamicity will lead to a decrease in chaperone activity. It seems that there is an ideal limit between the chaperone’s accessibility to the client protein and the chaperone’s structural dynamics. According to DLS results (Figure 3A), we found that the need to increase the assembly temperature (60 °C) is obvious. When the BαP molecular population was examined in the fresh state, almost two major populations were observed (74 and 297 nm), while when the temperature increased up to 60 °C, this population reassembled and changed its virtually single population (220 nm), which assured that all AuNC-containing particles were encapsulated. Although two additional populations with smaller sizes, 26 and 93 nm, appeared when the temperature of the mixture cooled back (Figure 3A), the most abundant and also the largest size population, 220 nm, is likely to be the main BαP. This population remained in the same size as that at 60 °C and was consistent with another remarkable result that as the system cooled down, there was no decrease in the fluorescence of BSA–AuNC (Figure 3B). These implied that the phase transition of PNIPAM does not change the core BαP assembly, and the linearization of PNIPAM may simply occur to the diffused outer layer or non-AuNC-containing PNIPAM particles.

The mechanism leading to BαP–Si stabilization may be attributed to increased stabilization of AuNC capping ligands or reduced particle interactions. First, the photoemission of AuNCs is mainly affected by the capping ligand such as certain thiol residues of BSA.^55^ We have tested and confirmed that α-Crys could not be used to synthesize AuNCs, indicating that α-Crys is not likely to contribute capping ligands of AuNCs regardless of two cysteine residues with αA-Cry (αB-Cry does not have cysteine residue).^28^ We thus suspect that the silica shell may play a role in directly or indirectly affecting the capping ligand stability. Direct silica interaction with BSA may promote capping ligand exchange by active silanol of TEOS or the amine group of APTES, leading to stronger photoemission of B–Si at high temperatures (Figure 1A). Silica interaction with α-Crys at high temperatures may disrupt the assembly of the chaperone–client system of Bα and thus disrupt the capping ligand structure contained in the client protein (BSA), leading to complete fluorescence quenching of Bα–Si (Figure S5). In contrast, the compressed/squeeze property of the PNIPAM polymer at high temperatures may reduce the silica interaction and enhance the wrapping of α-Crys to BSA–AuNC, leading to increased thermal stability of BαP–Si at high temperatures (Figure 4). Second, based on FTIR results, we suspect the partially exposed wrapping molecule BSA in B–Si could accelerate particle aggregation at high temperatures, causing instability. High temperatures above 600 °C lead to the decomposition of silica-encapsulated BSA–AuNCs and the transformation of gold nanoclusters into gold nanoparticles.^20^ Our data suggested a thermal (>37 °C) stress-induced photoemission increase accompanied by structural fragmentation/reorganization of B–Si (Figure 1), which may eventually lead to aggregation and formation of gold nanoparticles. For BαP–Si, the partially exposed PNIPAM (Figure 4C) could be condensed on the silica surface as it undergoes a linear-to-globular transition and can thus be more inert toward temperature-induced aggregation or other interactions. However, the decrease in fluorescence may be attributed to light scattering. Once encapsulated by silica, the size of BαP–Si is stable as shown by TEM (Figures 5 and S6B). In contrast, the size of conventional B–Si changes at high temperatures (the TEM image is shown in Figure 1). For this case, it is reasonable to suspect that the change of fluorescence may also be attributed to changes in light scattering in addition to the change of capping protection. It should also be noted that the shape and surface characteristics of nanoparticles further contribute to fluorescence properties, highlighting the intricate interplay between components in nanosystem design.

## Conclusions

Here we explored the impact of silica encapsulation-induced physicochemical stresses that have been overlooked by many applications and proposed a new nanoassembly to prolong the application window for cell imaging. Moreover, we established the role of α-Crys and PNIPAM in protecting BSA–AuNCs against the stresses. Although the role of α-Crys in supporting BSA–AuNC against thermal stress was evident, in the presence of the silica shell, this role was completely lost. Thus, the silica coating strategy to increase the stability of fluorescence emission of some biomacromolecule-based fluorophores cannot be predicted due to the complicated higher-order structure and strong silica interaction. When PNIPAM was added to the BαP complex, due to the barrier effect between α-Crys and the silica shell, the destructive effect of silica on the chaperone’s protective role disappeared. In addition, PNIPAM facilitates the accessibility of proteins and exhibits dynamic behavior crucial for chaperone function. These results advance our understanding of the complexity relevant to protein chemistry, polymer science, and nanotechnology, emphasizing the need for an approach to balance various parameters for optimal performance. Although long-term stability studies under more physiologically relevant conditions would be necessary to provide a more precise evaluation with a comprehensive understanding of the nanosystem’s behavior, the use of nontoxic materials (α-Crys and PNIPAM) should lower the concern of toxicity risk for potential therapeutic utility.

## Supporting Information Available

Materials and results: fluorescence intensity of BSA–AuNCs, investigating the synthesis of BSA–AuNC at 60 °C; TEM and fluorescence intensity of B–Si fabricated using 5× molar concentration of TEOS; SDS-PAGE patterns of different fractions of purified α-Crys; TEM and fluorescence intensity of the encapsulation of BSA–AuNC-α-Crys (Bα) by Si (Bα–Si); FTIR spectroscopy of B–Si and the TEM photograph of BαP–Si at 37 °C for 24 h; protein release pattern of whole proteins (BSA–AuNC and α-Crys) from BαP–Si; QY measurement for BSA–AuNC, B–Si, and BαP–Si; cell studies; BET and BJH pore size distribution patterns of mesoporous silica nanoparticles and BαP–Si; and TEM size distribution (PDF)
