# Supplementary material for: Chaperone/Polymer Complexation of Protein-Based Fluorescent Nanoclusters against Silica Encapsulation-Induced Physicochemical Stresses
Source: Biomacromolecules. 2024 Sep 17;25(10):6515–25. doi: 10.1021/acs.biomac.4c00689 (PMC11480988; doi:10.1021/acs.biomac.4c00689)
Supplement: Supplementary file 1 — bm4c00689_si_001.pdf [file bm4c00689_si_001.pdf]

Chaperone/Polymer Complexation of Protein-based Fluorescent Nanoclusters Against Silica Encapsulation-Induced Physico-chemical Stresses

Mohsen Akbarian[1,3], I-Ni Chen[1], Pei-Hsuan Lu[1], Quynh-Trang Do[1], Shun-Fen Tzeng[2], Ho-Hsuan Chou[1], and Shu-Hui Chen[1]*

Department of Chemistry[1], Department of Life Science[2], National Cheng Kung University, Tainan, Taiwan
[3]Marquette University School of Dentistry, Milwaukee, Wisconsin 53233, USA
*shchen@mail.ncku.edu.tw

1. **Supporting Methods**

1.2. Extraction and purification of bovine lens α-Crystallins

As published before,[1] the total soluble lens proteins (TSPs) were extracted from the bovine eye lens. To sum up, lenses were dissected from the eyeballs of bovines and a 10% (W/V) homogenate was prepared in 25 mM Tris (pH 8.0), containing 0.5 mM EDTA, 0.1 M NaCl, 10 mM β-mercaptoethanol (β-ME), and 0.01% NaN_3. Then the TSPs, as supernatant, were obtained by the centrifugation of the homogenate at 12000 g, for 20 min at room temperature. For the next step, the solution of TSPs was dialyzed in extensive water and dried by the freeze-dryer. Then, the powder of TSPs was solubilized (40 mg/mL) in Tris buffer (25 mM, pH 8.0 containing 100 mM NaCl) and loaded into a Sephacryl S-200 matrix (Sigma Aldrich, United States) (45 × 1 cm column). The volume of the loaded protein solution was chosen equal to 1.3 % (V/V) of the total column matrix. Then, running buffer (25 mM, pH 8.0 containing 100 mM NaCl) was used to elute protein fractions. The flow rate was selected at 200 µL/min with a fraction size of 2.0 mL. In the end, the quality of the purification of α-Crys was shown using denaturing-reducing 12% SDS-PAGE (SE 250 Minivertical Unit, Cytiva, USA).[2] Fractions containing pure α-crystallins (α-Crys) were identified, collected, dialyzed against water and finally lyophilized.

1.3. The polymerization of N-Isopropylacrylamide

N-Isopropylacrylamide monomer was used for the synthesis of NIPAM polymers (PNIPAM). For the polymerization, 1.0 g of the monomer was solubilized completely in 50 ml of pure dioxane, then 10.1 mg of AIBN as a catalyzer was added and the resultant mixture was refluxed at 80 ºC for 6.0 hrs. Finally, the obtained PNIPAM was dialyzed several times against distilled water by a dialysis tube (Spectra/Por®/cut-off 3.5 kDa), and the polymer was frozen and dried with a lyophilizer.

1.4. BSA-AuNC synthesis

According to a standard method,[3] at first, 50 mg/mL BSA was prepared in an aqueous solution and mixed well with 5 mL of 10 mM $HAuCl_4$. Then, the pH of the mixture was raised to 12 with NaOH and the final mixture was incubated for 12 hrs in an incubator at 37 °C with gentle shaking. After synthesizing the gold and protein complex, the obtained final product was stored at 4 °C for further use.

1.5. Synthesis of B-Si

B-Si was synthesized following the published method[4] with modifications. Briefly, a volume of 75-100 μL of BSA-AuNCs the stock solution was added with 5 mL ethanol and 200 μL of ammonia (28%), and 40 μL of TEOS. After 24 hrs of incubation at room temperature, the obtained precipitate was separated by centrifugation and washed at least three times in distilled water.

1.6. High-resolution transmission electron microscopy

High-resolution transmission electron microscopy (HR-TEM) (JEM-2100F, Joel) was used to determine a general view of the structure of BαP-Si nanoparticle and its internal elements. Thus, the samples (1.0 mg/mL in deionized water) were completely dispersed for 5 min with a sonicator, and a 20 μL was incubated on the absorbent surface of the 300-mesh carbon support grid (copper mesh) for 5 min. Then, with the help of filter paper, the solution was absorbed and the rest was dried overnight. The finalized nanoparticle sample (BαP-Si) and PNIPAM complex with chaperone and BSA-AuNC (BαP) were prepared for HRTEM. Also, to check the distribution of gold clusters as well as carbon, silicon, oxygen and nitrogen atoms in the samples, the elemental map was used.

1.7. Dynamic light scattering

Dynamic light scattering (DLS, DelsaNano C, Beckman Coulter Inc, Brea, CA, USA) was used to determine the hydrodynamic diameter of particles. Correspondingly, the interaction between the α-Crys chaperone and the target protein (BSA-AuNC), as well as the interaction between the PNIPAM and the proteins complex were investigated with this assay. Hence, 2.0 mg/mL (in distilled water) of samples were prepared and their particle sizes were measured at two sets of temperatures (25 °C and 60 °C). To present the results, a bar chart containing the average particle size was used, which can show the changes in particle size more simply.

1.8. Functionalization with 17α-ethynylestradiol (EE2)

MCF7 cells express several estrogen receptors (ER+), making them responsive to estrogenic compounds such as 17α-ethynylestradiol (EE2).[5,6] To bind BαP-Si nanocluster to EE2, the linker (2,5-Dioxopyrrolidin-1-yl 2-(2-(2-(2-azidoethoxy)ethoxy)ethoxy)acetate) containing activated carboxyl group was synthesized in house.[7] Due to quenching susceptibility, EE2 conjugation was done with the starting of coupling the linker to EE2. Briefly, a mixture (200 μL) containing EE2 (4mM), THPTA (2

mM), $CuSO_4$ (1 mM), sodium ascorbate (20 mM), and the linker (150 μM) was shaken at room temperature for 2 hrs. The resulting linker-EE2 was extracted by ACN (100 μL) and dried by a speed-vac concentrator. For conjugation, the BαP-Si (1.0 mg) was dissolved in water and then added with the linker-EE2 (150 μM). The resulting mixture was shaken at room temperature for 24 hrs. The EE2-labeled BαP-Si was collected by centrifugation (10000 g, 5 min), washed three times with deionized water, and dried.

1.9. Cell culture and fluorescence microscopy.

To start the cell study, the breast cancer cell line (MCF-7) in RPMI 1640 culture medium (containing 10% (v: v) FBS, 1% Pen/Strep, under 5% CO_2, and 90% humidity) at 37 °C was cultured. Then, 10,000 MCF-7 cells were seeded in each well (24 flat-well plate) with a glass coverslip in the bottom (12×0.11mm). After overnight incubation in the defined conditions (80% confluence of the cell), the cells were first washed with phosphate-buffered saline and then incubated with the culture medium containing 50 μg/mL of samples BαP-Si-EE2, linker- BSA-AuNC and BSA-AuNC-EE2, separately, for 2.0 hrs and 24 hrs. At each interval, the cells were first washed with phosphate-buffered saline and stained with 4', 6-diamidino-2-phenylindole (DAPI) dye. For staining, the dye was prepared in phosphate-buffered saline (1.0 mg/mL) and each well was incubated with the DAPI solution (10 μL) for 15 min. After two washing steps with phosphate-buffered saline, the cells were fixed with 4% paraformaldehyde solution for 10 min. Imaging of cells was done with a fluorescence microscope by considering 405 nm (DAPI filter) and 635 nm laser lines.[8] The fluorescence studies were performed using a Jobin Yvon/HORIBA HR320 fluorimeter. Fluorescence microscopy was applied by an Olympus FluoView fluorescence microscope (FV1000, Tokyo, Japan) and lyophilizing was done by a PANCHUM (Taiwan) freeze dryer.

1.10. In vitro protein release study

To study the release of proteins (BSA and α-Crys) from BαP-Si, 1.0 mL of PBS solution containing 10 mg of nanoparticles was prepared. At the end of each time point incubation at 37°C with continuous/rigorous shaking, the solution was centrifuged (10 min at 12,000 g), 0.1 mL of the supernatant was removed, and the same volume of the buffer was replaced again to maintain the sinking

conditions. The number of proteins in all obtained supernatants was determined by Lowry assay. Then, following formula (1) cumulative release was plotted.

$$Cumulative\ proteins\ release = \frac{C_i \times 1 + \sum_n^{n-1} C_{i_\times} 0.1}{W} \quad \mathbf{(1)}$$

where W is the initial amount of proteins (BSA and α-Crys) in the nanosystem C is the concentration of the released protein at the time i.

1.11. Attenuated total reflection Fourier transform infrared spectroscopy

Attenuated total reflection Fourier transform infrared spectroscopic (ATR-FTIR, RX-1 FT-IR, PerkinElmer, Shelton, USA) analysis was performed using ~ 10 mg of each B-Si and BαP-Si at a wave number between 400 and 4000 cm^{-1}.

1.12. Brunauer–Emmett–Teller analyses

The solid Si and BSA-AuNC were subjected to Brunauer–Emmett–Teller (BET) analyses. The adsorption analyses were made with a volumetric adsorption analyzer (TriStar II 3020 V1.03). First, the samples were degassed and the analysis bath temperature was considered -195.850 °C. Then the N2 adsorption and desorption and the isotherms were obtained in the pressure range P/P0 = 0.001- 0.995.

2. Supporting results

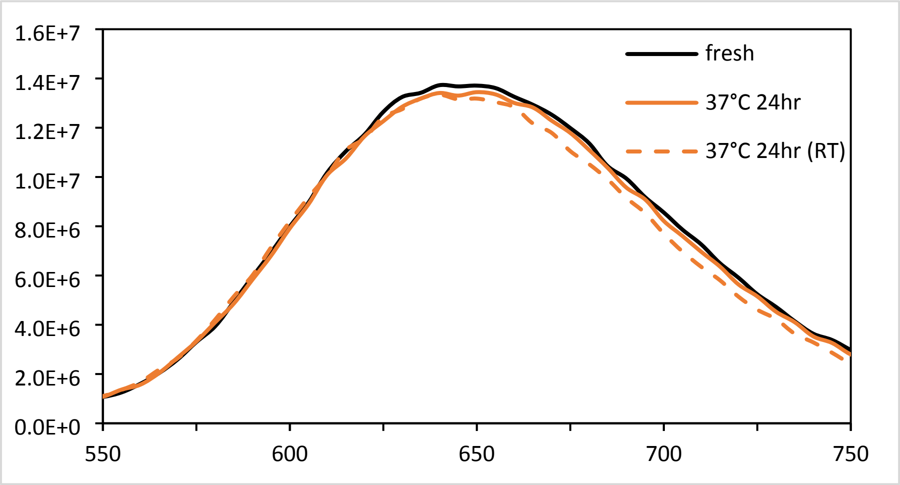

Figure S1. Fluorescence intensity of BSA-AuNCs.

A

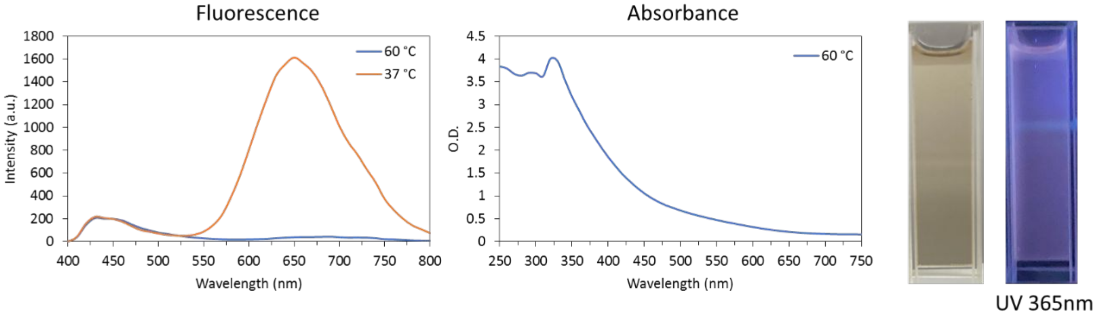

B

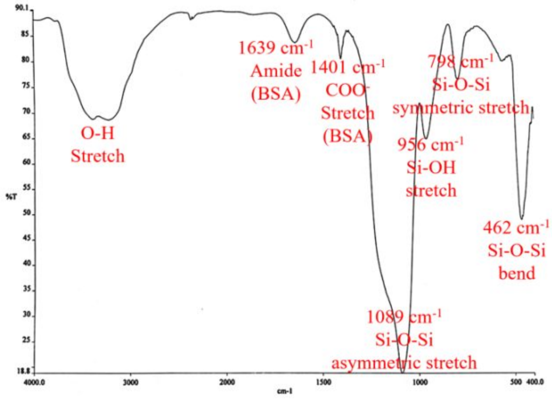

Figure S2. A) Investigating the synthesis of BSA-AuNC at 60 °C. Similar to the method that was done for the synthesis of BSA-AuNC at 37 °C, the temperature of 60 °C was considered as different synthesis pot. Although the absorbance of the synthesized product at 60 °C has increased in the 330 nm, compared to the same concentration of BSA-AuNC synthesized at 37 °C, no recordable fluorescence was observed. The

photographs taken of the BSA-AuNC at 60 °C also show that no fluorescence emission can be seen under the excitation in the UV region (365nm). **B)** FT-IR of B-Si

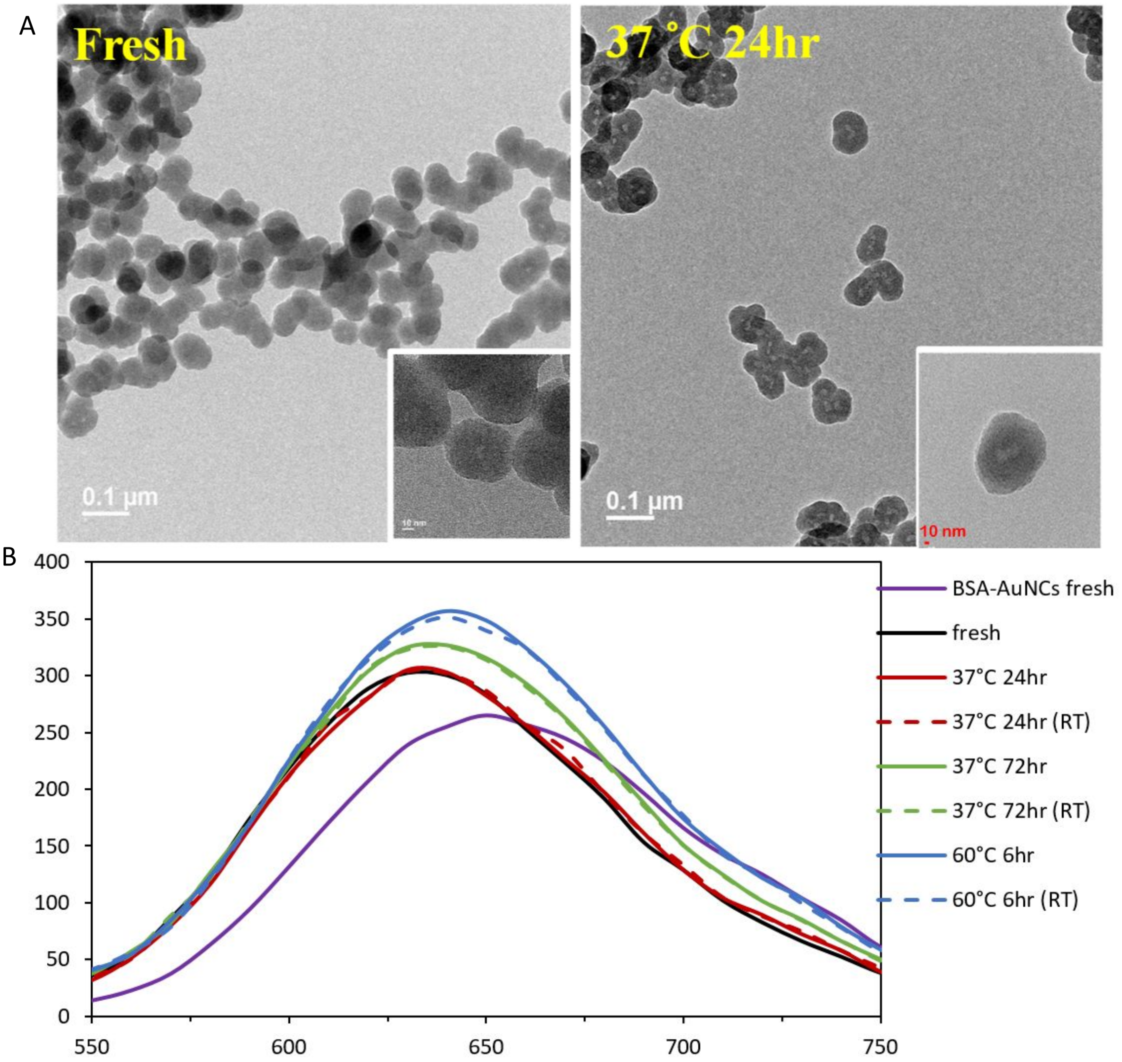

Figure S3. A) TEM and **B)** fluorescence intensity of the B-Si fabricated using 5× molar concentration of TEOS.

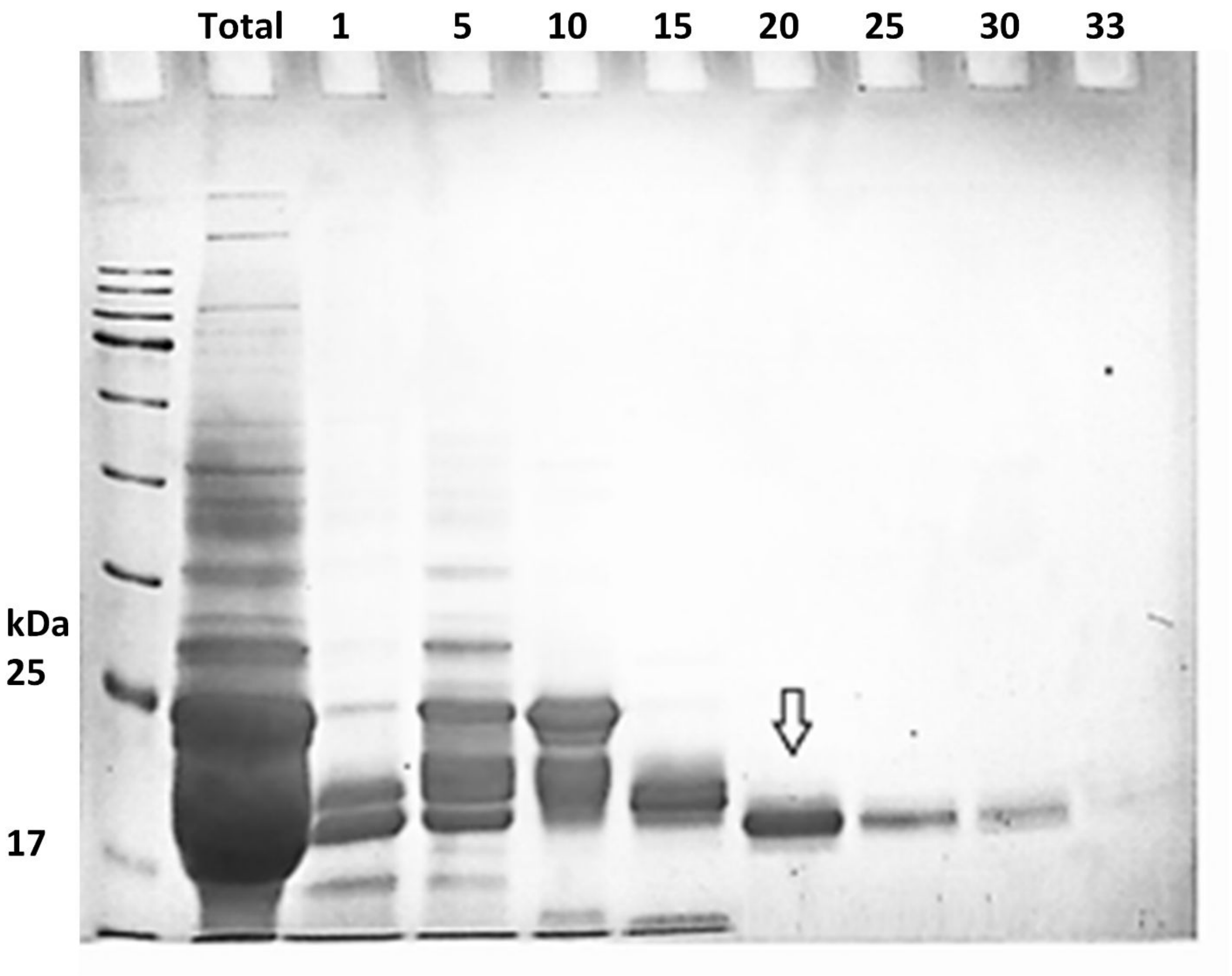

Figure S4. SDS-PAGE patterns of different fractions of eluted proteins from Sephacryl S-200 column. αA- and αB-Crys have controversial molecular sizes. In most studies, the size between 19-20 kDa have been mentioned about them. The arrow shows the purified fraction with the molecular size near 20 kDa.

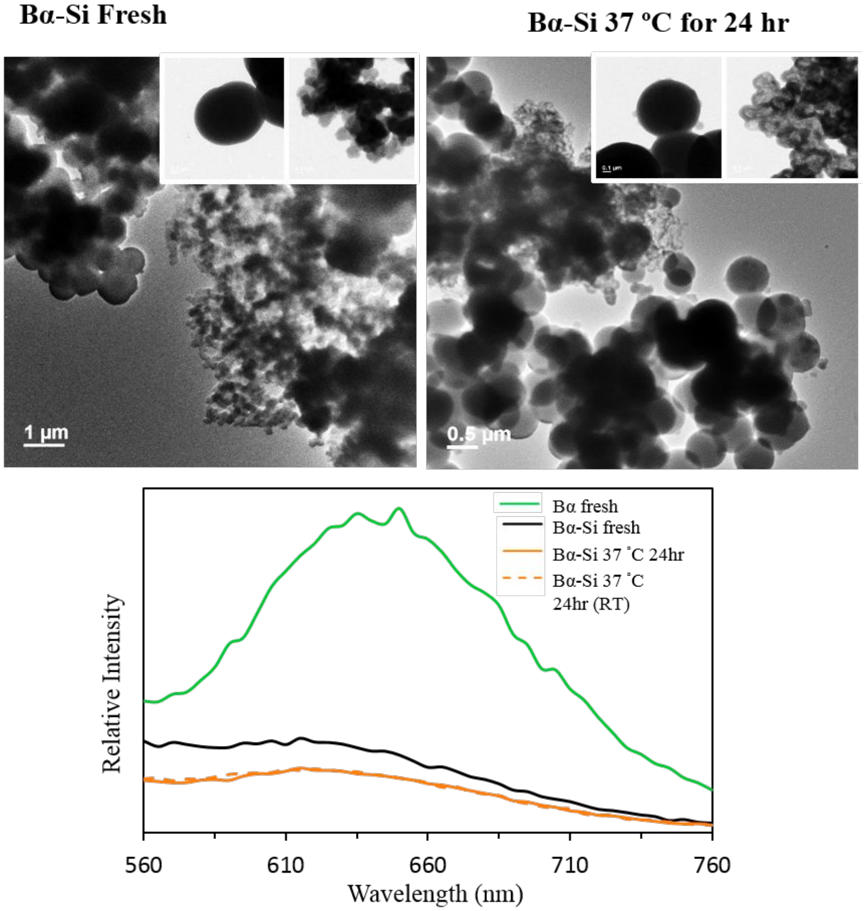

Figure S5. TEM and fluorescence intensity of the encapsulation of BSA-AuNC-α-Crys (Bα) by Si (Bα-Si).

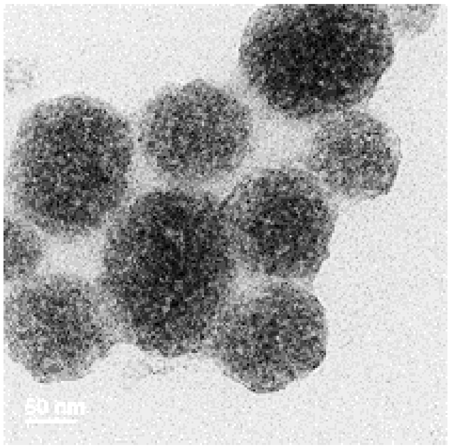

Figure S6. TEM photograph of BαP-Si at 37 ºC for 24 hrs.

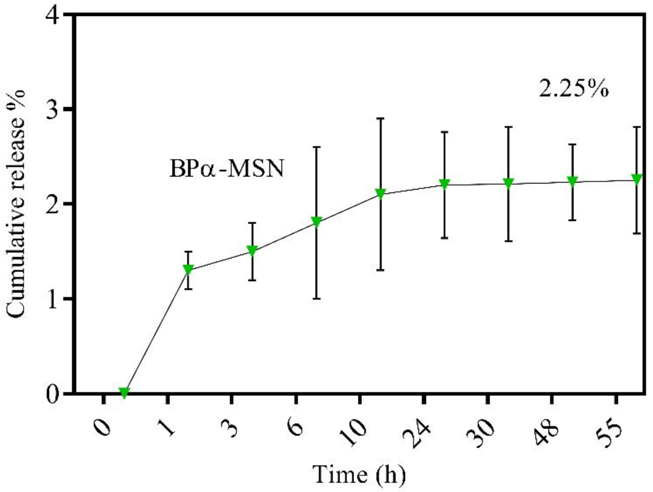

Figure S7. Protein release pattern of whole proteins (BSA-AuNC and α-Crys) form BαP-Si.

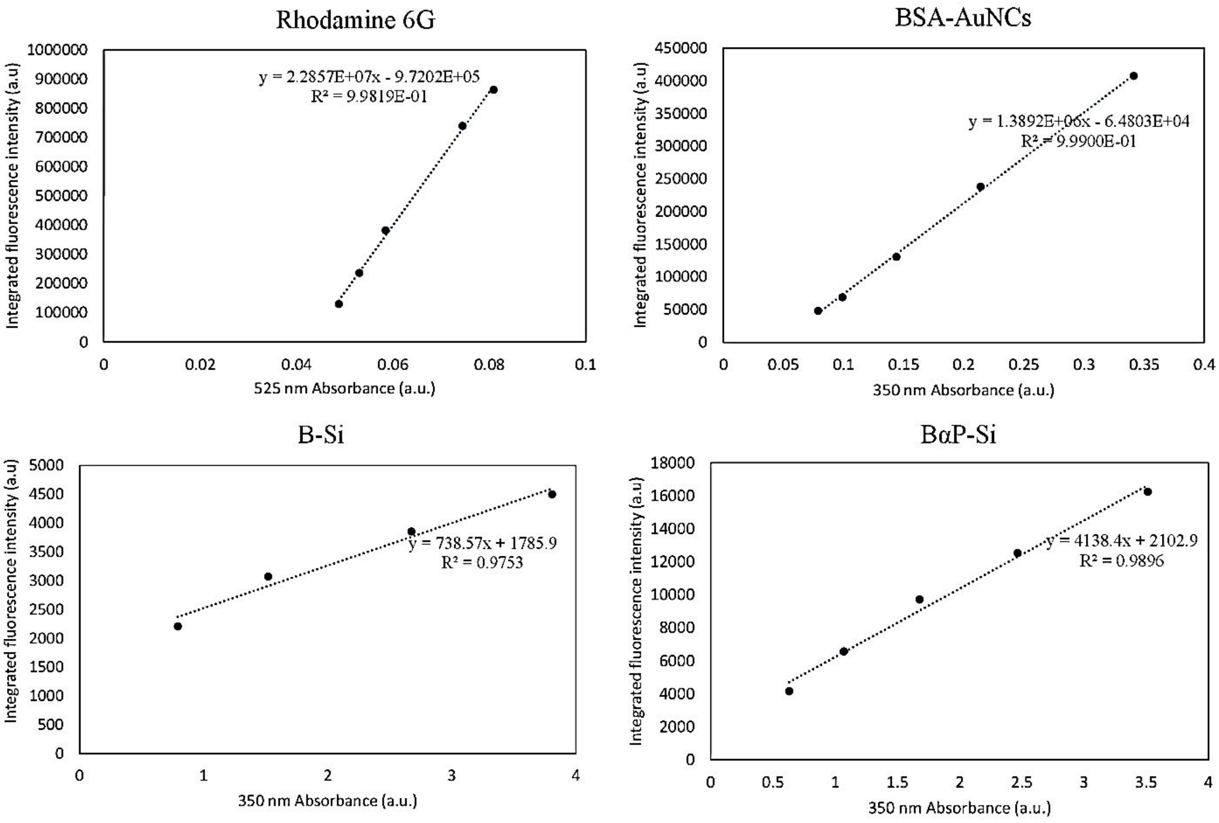

Figure S8. Quantum yeild (QY) measurment using rhodamin 6G (QY 95%) as the reference. QYs (95% x slope of the particles/slope of rhodamin 6G) were calculated to be 5.77% (BSA-AuNCs), 0.003% (B-Si), and 0.017% (BαP-Si), respectively.

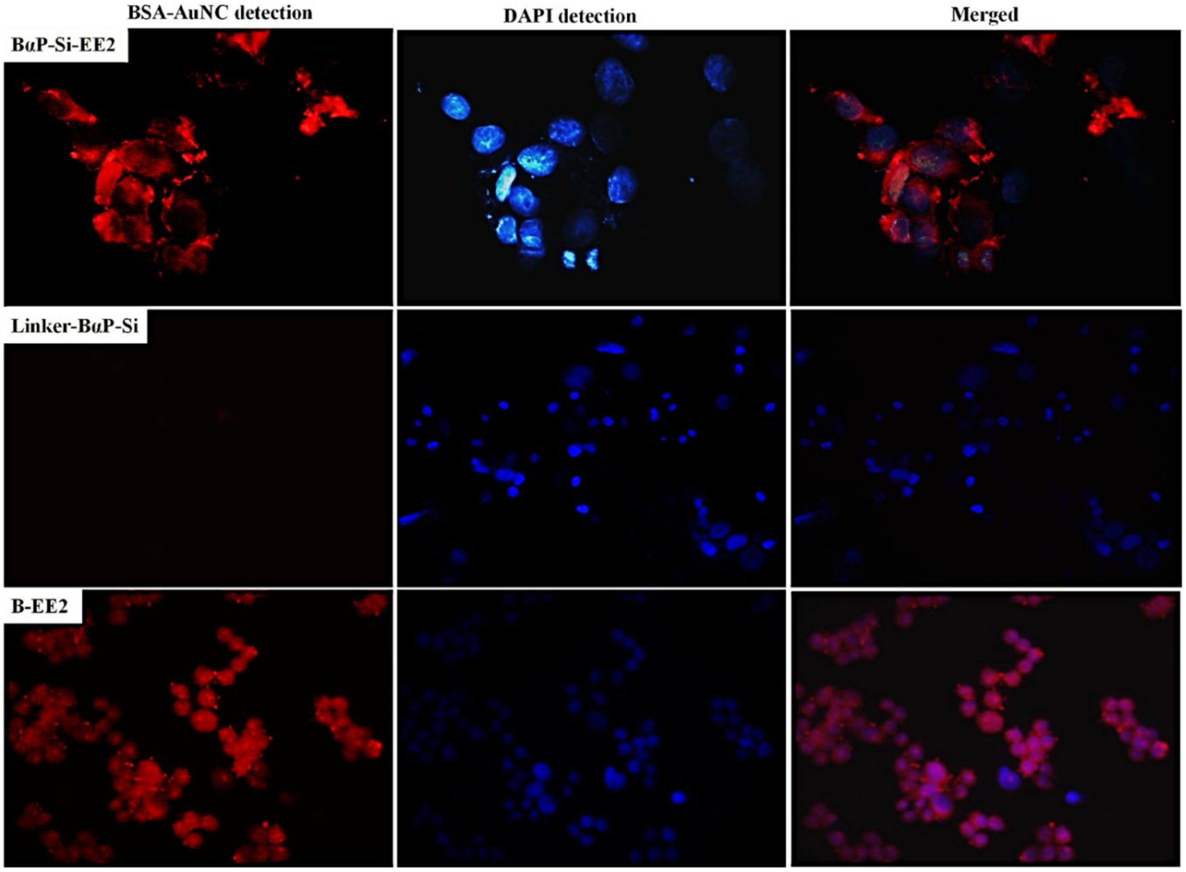

Figure S9. In vitro assessment of cell binding activity of the EE2 targeted BαP-Si nanoparticle.

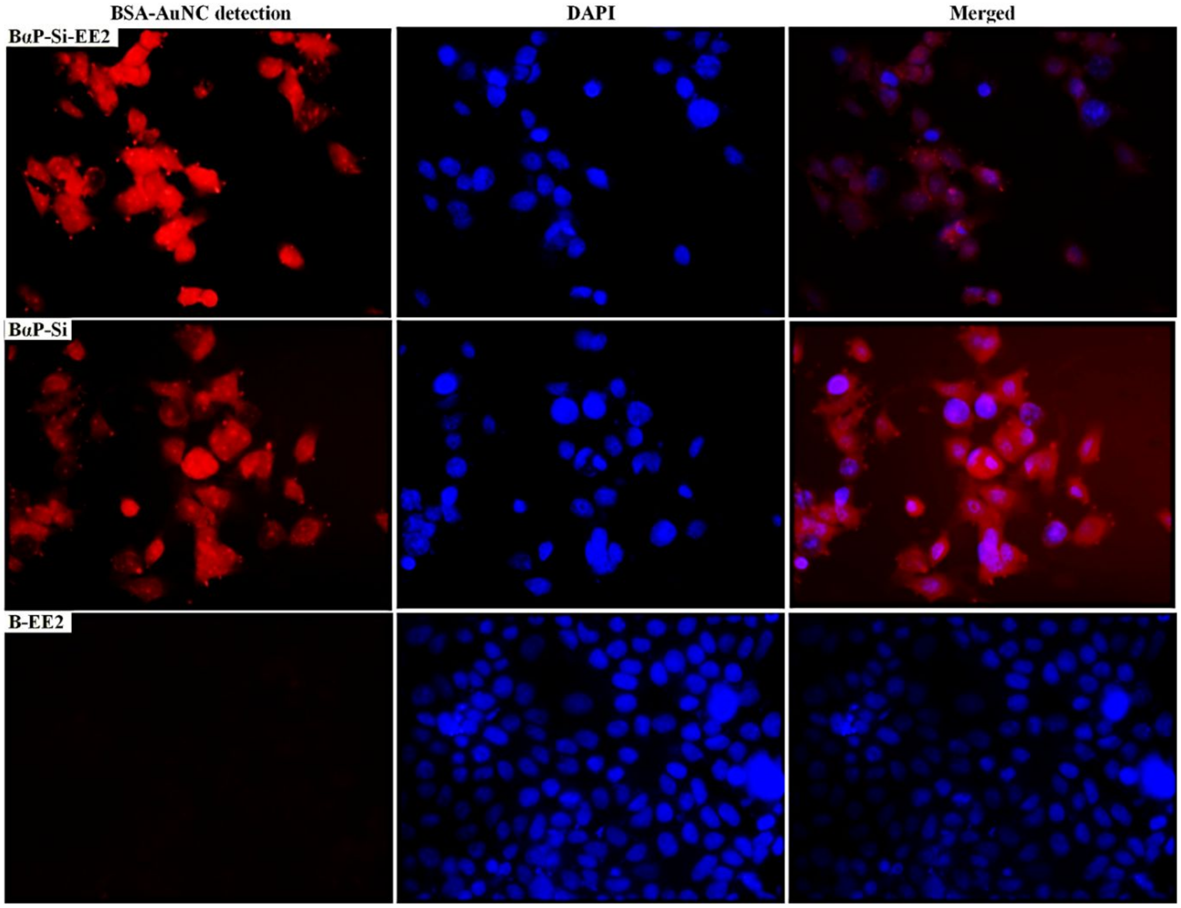

Figure S10. 24 hrs incubation of the different applied particles with MCF7 cells.

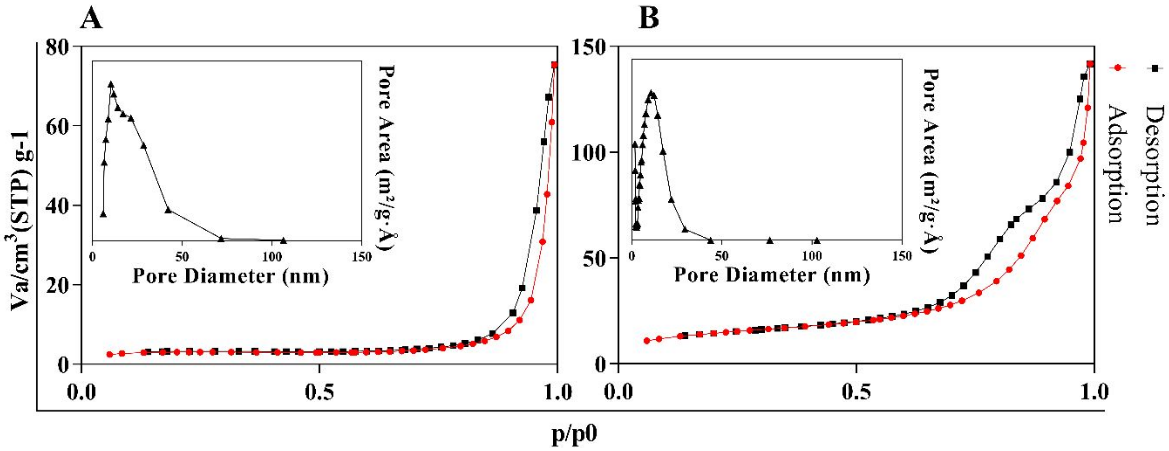

Figure S11. BET and BJH pore size distribution patterns of bare mesoporous silica nanoparticles (MSN) (A) and BαP-Si (B). MSN was synthesized by using CTAB template.[8] Comparing A and B, they show that in BαP-Si sample, although without applying CTAB template, the pore sizes were evidenced in the range of meso-size with more monodispersity.

Table S1. The obtained physical parameters of the studied BαP-Si.

Nanoparticles	Adsorption average pore width (4V/A by BET) (nm) [8]	BJH Adsorption average pore diameter (4V/A)	BJH Desorption average pore diameter (4V/A)	BET surface area ($m^2\ g^{-1}$)	Total pore volume ($m^3\ g^{-1}$)
MSN	10.53248	44.3167	36.4872	945.19 +02	0.24888
BαP-Si	10.24210	14.3730	14.2271	508.019 +02	0.130079

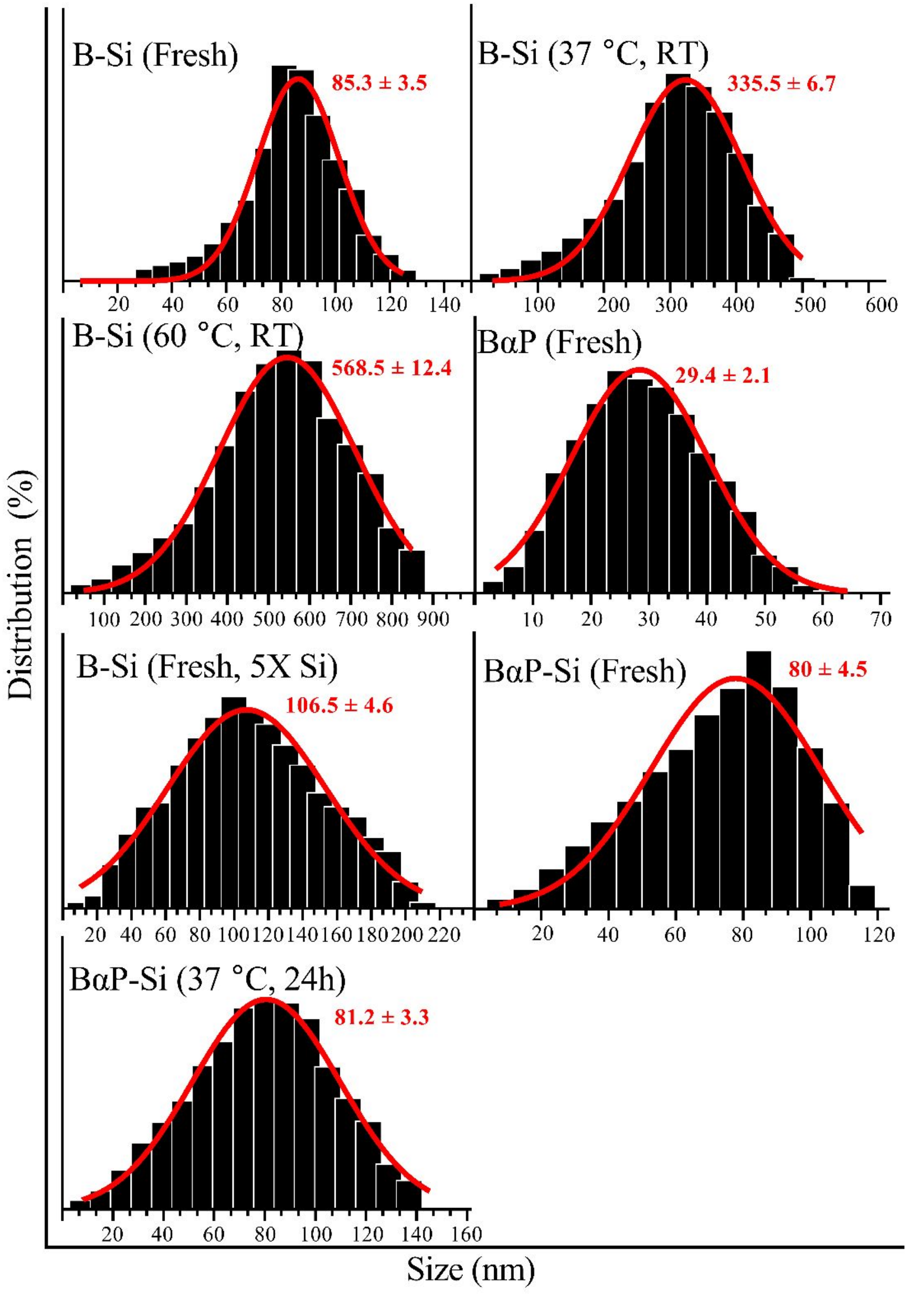

Figure S12. Size distribution of different nanoparticles applied in this study. Using ImageJ software (ij154-win-java88) the corresponding TEM images were used to calculate the size distributions.

References

1. Khoshaman, K.; Yousefi, R.; Tamaddon, A. M.; Abolmaali, S. S.; Oryan, A.; Moosavi-Movahedi, A. A.; Kurganov, B. I., The impact of different mutations at Arg54 on structure, chaperone-like activity and oligomerization state of human αA-crystallin: The pathomechanism underlying congenital cataract-causing

mutations R54L, R54P and R54C. *Biochimica et Biophysica Acta (BBA)-Proteins and Proteomics* **2017,** *1865* (5), 604-618.

2. Laemmli, U. K., Cleavage of structural proteins during the assembly of the head of bacteriophage T4. *Nature* **1970,** *227* (5259), 680-685.

3. Xie, J.; Zheng, Y.; Ying, J. Y., Protein-directed synthesis of highly fluorescent gold nanoclusters. *J. Am. Chem. Soc.* **2009,** *131* (3), 888-889.

4. Le Guével, X.; Hötzer, B.; Jung, G.; Schneider, M., NIR-emitting fluorescent gold nanoclusters doped in silica nanoparticles. *Journal of Materials Chemistry* **2011,** *21* (9), 2974-2981.

5. Van den Belt, K.; Berckmans, P.; Vangenechten, C.; Verheyen, R.; Witters, H., Comparative study on the in vitro/in vivo estrogenic potencies of 17β-estradiol, estrone, 17α-ethynylestradiol and nonylphenol. *Aquatic toxicology* **2004,** *66* (2), 183-195.

6. Tsai, C.-Y.; Li, C.-W.; Li, J.-R.; Jang, B.-H.; Chen, S.-H., Steroid probes conjugated with protein-protected gold nanocluster: specific and rapid fluorescence imaging of steroid receptors in target cells. *Journal of fluorescence* **2016,** *26* (4), 1239-1248.

7. Huang, W.-C.; Huang, L.-J.; Hsu, L.-S.; Huang, S.-T.; Lo, W.-T.; Wang, T.-F.; Sun, W.-T.; Wei, W.-Y.; Lee, Y.-S.; Chuang, S.-H., Selective and predicable amine conjugation sites by kinetic characterization under excess reagents. *Scientific reports* **2021,** *11* (1), 21222.

8. Akbarian, M., et al., *Theranostic mesoporous silica nanoparticles made of multi-nuclear gold or carbon quantum dots particles serving as pH responsive drug delivery system.* Microporous and Mesoporous Materials, 2022. **329**: p. 111512.
